# Intention to Get COVID-19 Vaccinations among Ophthalmology Residents in Poland: A Cross-Sectional Survey

**DOI:** 10.3390/vaccines9040371

**Published:** 2021-04-11

**Authors:** Joanna Konopińska, Iwona Obuchowska, Łukasz Lisowski, Natalia Dub, Milena Kozera, Marek Rękas

**Affiliations:** 1 Department of Ophthalmology, Medical University of Bialystok, 15-276 Bialystok, Poland; iwonaobu@wp.pl (I.O.); lisowski@vp.pl (Ł.L.); natalkand@wp.pl (N.D.); 2 Department of Ophthalmology, Military Institute of Medicine, 04-141 Warsaw, Poland; m.kozera@onet.eu (M.K.); rekaspl@gmail.com (M.R.)

**Keywords:** COVID-19 vaccine, vaccine acceptance and hesitancy, ophthalmology residents, Poland

## Abstract

This study aimed to evaluate the acceptability of coronavirus disease of 2019 (COVID-19) vaccination among ophthalmology residents in Poland. An online, self-administered, anonymous survey was distributed among Polish ophthalmology residents in early 2021. Of 126 residents who completed the survey, 71.4% indicated that they would get vaccinated, 17.5% were unsure, and 11.1% would refuse vaccination. Married respondents with children (*p* = 0.036) and respondents living with their families (*p* = 0.310) were more likely to accept vaccination, believing that the vaccine is effective (*p* = 0.002 and *p* = 0.001, respectively), and fearing for themselves (*p* = 0.031 and *p* = 0.023, respectively) or their families (*p* = 0.032 and 0.055, respectively) getting infected. Respondents who contracted COVID-19 often reported the expected relief in sanitization (*p* = 0.011) as their reason for vaccination, and the previous severe acute respiratory syndrome coronavirus 2 (SARS-CoV-2) infection (*p* = 0.050) as their reason for not vaccinating. Unmarried residents and residents living alone often declared that they were waiting for the effectiveness and long-term complications of the vaccine to be assessed (*p* = 0.005, both). Residents living with their families were significantly less likely to report COVID-19 as the reason for refusing vaccination (*p* = 0.022). In conclusion, most ophthalmology residents expressed a willingness to get vaccinated. Marital status and cohabitants affect vaccination acceptance. People with COVID-19 have different reasons for accepting or refusing vaccination. Medical authorities should persuade citizens more to vaccinate.

## 1. Introduction

On 11 March 2020, the World Health Organization (WHO) announced the severe acute respiratory syndrome coronavirus 2 (SARS-CoV-2) pandemic. In Poland, the first case of coronavirus disease of 2019 (COVID-19) was recorded on 4 March 2020, and on 27 December 2020, the first vaccination was performed to prevent the spread of SARS-CoV-2. However, before vaccination, there was a constant struggle with the COVID-19 pandemic, which has become a global threat to public health. This forced the health system to radically change the way health care is organized, including ophthalmology departments [1]. Ophthalmologists have been recognized to be at high risk of COVID-19 infection, due to close contact with patients. Consequently, scheduled ophthalmology advice and surgery during the pandemic have been significantly reduced, and only emergency ophthalmology care has been allowed.

Due to the development of the SARS-CoV-2 pandemic, many ophthalmology organizations and societies have developed recommendations for managing ophthalmology patients [1], and adequate recommendations have also been developed within other fields of medicine. However, healthcare workers—including residents—comprise most of the COVID-19-infected population and contribute to spreading the disease, both among their families and patients [2,3]. In Italy, one of the most affected countries during the first wave of the pandemic, 10% of infected patients were healthcare workers (HCWs) [4].

The widespread global SARS-CoV-2 pandemic, and the corresponding introduction of further lockdowns—as well as the viral mutations that led to new, more infectious strains—made COVID-19 vaccination the only hope to reduce the pandemic and return the public and service health sectors to their normal states. Many pharmaceutical companies have been racing to create COVID-19 vaccines that can be brought to the market as soon as possible and disseminated on a mass scale. Although most realistic predictions assumed that a new vaccine could not be developed within one year, the first COVID-19 vaccine was registered at the end of 2020, and initial vaccination began globally.

Although the COVID-19 vaccination program can significantly alleviate the problems associated with the spread of the disease, there are still many people who are skeptical about the vaccine, and despite its availability, refuse to take it. Most doubts are raised based on the fact that the vaccine was developed in a very short period of time, and its long-term effects and effectiveness remain unknown [5,6]. The WHO classified “vaccine hesitation” as one of the top 10 health threats in the world last year.

Taking this into account, in our work, we decided to assess the attitudes of ophthalmology residents in Poland toward vaccination against COVID-19, based on an anonymous survey. Resident doctors are a specific group of health workers. They are young, and therefore less likely to develop severe SARS-CoV-2 infection [7]. On the other hand, given their medical knowledge, they are more aware of not only the health risks of COVID-19, but also the possible adverse effects of the vaccine itself. Like other individuals, they are also tired of the constant epidemiological restrictions and limitations of social life. Moreover, detailed studies have shown that the vast majority of ophthalmologists in training (81–93.8%) believe that the pandemic negatively affected their ophthalmology training [7,8,9,10,11,12].

This study aimed to analyze the motivations of ophthalmology residents to get vaccinated and why they are reluctant to take the COVID-19 vaccine, based on an anonymous survey. To the best of our knowledge, this is the first study to address the attitude of ophthalmology residents toward SARS-CoV-2 vaccination. Understanding the attitude of young doctors toward COVID-19 vaccination and learning about the sources from which young doctors obtain information on this subject, will enable medical authorities to plan appropriate steps to successfully implement a large-scale vaccination program.

## 2. Methods

This study was approved by the Bioethics Committee of the Medical University of Bialystok (no. APK.002.87.2021), and was in accordance with the 1964 Declaration of Helsinki, its later amendments, or comparable ethical standards. We used an anonymous questionnaire, designed by all authors, that was created on Google Forms and distributed by email and/or Facebook and WhatsApp messengers in January 2021, with a response time of one week. We used the residents’ University and/or hospitals’ emails, and official websites and social media profiles (Facebook) of the following institutions: Medical University of Bialystok and Department of Ophthalmology, Military Institute of Medicine in Warsaw. Moreover, ophthalmology residents were invited to participate in the study through a link to the survey that was delivered via the social media profile of “National Consultant of ophthalmology Prof. Marek Rękas”, which gathers Polish ophthalmology workers.

The questionnaire contained 15 closed-ended, single-answer, and multiple-choice questions. Initially, a pilot study was conducted on a group of 12 residents to optimize the survey in terms of clarity, question validity, and time necessary to complete the survey. The participatory pilot survey involved informing the respondents that they were in the pre-test phase. The respondents were asked items that would be answered as part of the questionnaire; specifically, they were asked for their reactions, comments, and suggestions. The authors asked respondents about how clear the instructions were, and which questions were hard to answer [7].

The survey was divided into two sections. The first section collected respondent demographic data, including gender, marital status, place, and residence conditions. Residents were also asked if they were previously infected with COVID-19, were in quarantine, or worked with SARS-CoV-2-positive patients. The second section assessed the attitudes of residents toward the COVID-19 vaccination, including their reasons for accepting or refusing vaccination.

Participation in the survey was completely anonymous and voluntary. One question in the survey obtained the respondents’ consent to the statistical analysis of anonymous data, and their use for scientific publication. The exact model of the survey is available in the Supplementary Materials (see Supplement). When determining sample size, we assumed that 65% of responders in the non-COVID-19 working group would be willing to take the COVID-19 vaccine; thus, after applying a continuity correction, the study would require a sample size of 42 individuals in each group (i.e., a total sample size of 84, assuming equal group sizes) to achieve a power of 80%, which was sufficient for detecting differences in proportions of 0.25 between the two groups (the COVID-19 working vs. the non-COVID-19 working group) using a two-sided *p*-value of 0.05.

### Statistical Analysis

Statistical analyses were performed using the R program, version 3.5.1 (R Foundation for Statistical Computing, Vienna, Austria.)

The studied variables were presented using basic descriptive statistics according to the measuring scale. The nominal variables were compared between groups using the chi-square test or Fisher’s exact test. The normality of the distribution of the quantitative variables was assessed using the Shapiro–Wilk test, data skewness and kurtosis indicators, and the visual assessment of histograms. The variance equality was checked using the Leven test. The quantitative variables measured between the two groups were compared using Student’s t-test or the Mann–Whitney U test. The three groups were compared using the analysis of variance test paired with the Tukey post-hoc test, or the Kruskal–Wallis test paired with the Dunn post-hoc test. The mean/median differences (MDs) with 95% confidence levels were also calculated, as appropriate. A multivariate logistic regression analysis, including sex, marital status, living situation, place of living, and hospital ward as predictors, was conducted to identify the variables significantly impacting willingness to take a COVID-19 vaccine. A *p*-value of <0.050 was considered statistically significant.

## 3. Results

A total of 126 ophthalmology residents participated in this study; of these residents, 102 (81%) were female and 24 (19%) were male. The sociodemographic characteristics of the study participants are shown in Table 1.

Among respondents, 90 (71.4%) declared their willingness to take the COVID-19 vaccine, 22 (17.5%) were unsure, and 14 (11.1%) would refuse the vaccination. Married residents with children were significantly more willing to take the COVID-19 vaccine than those without children (82.6% and 55.6%, respectively, *p* = 0.036); moreover, residents living with their family/partner were also more willing to take the vaccine than residents living alone (78/72% and 57%, respectively, *p* = 0.310). There were no statistically significant differences in vaccine acceptability when the subjects were compared based on sex, place of residence, COVID-19 incidence, having stayed in quarantine, or working in units treating COVID-19 patients.

Respondents who were willing to be vaccinated reported a fear of relatives and family getting infected (92.2%), a faith in the effectiveness of the vaccine in controlling the pandemic (77.8%), a fear of being infected (70%), and a willingness to set a good example for others (68.9%) as the main reasons for vaccine acceptability. Other less important reasons included the possibility of benefiting from the reduced epidemiological regime allowed for vaccinated individuals (43.3%), and a lack of fear of the vaccine’s adverse effects (25.5%). Respondents who contracted COVID-19 were significantly more likely to indicate that their vaccine acceptability was due to the expected sanitary relief, compared to those who did not contract COVID-19 (53.8% and 22.2%, respectively; *p* = 0.011). Married/cohabiting respondents with children were more likely to indicate that the vaccine was effective, compared to those without children or not in a relationship (76.1%, 37%, and 47.2%, respectively; *p* = 0.001), and feared contamination (65.2%, 33.3%, and 45.3%, respectively; *p* = 0.023); these factors indicated arguments in favor of accepting vaccination. Compared to those living alone, residents living with their family or partners more often gave the following reasons for accepting vaccination: belief in the effectiveness of the vaccine (*p* = 0.002), fear of COVID-19 infection (*p* = 0.031), fear of their family becoming infected (*p* = 0.032), and the willingness to set a good example for others (*p* = 0.027). There were no significant correlations between the reasons for vaccine acceptability and gender, place of residence, having been in quarantine, or working with COVID-19 patients.

The most common reasons for refusing COVID-19 vaccination were a willingness to wait for the effectiveness and long-term adverse effects of the vaccine to be assessed (72.2%), a feeling that the vaccine was not sufficiently tested (58.3%), a fear of complications (41.7%), and a belief that having already contracted COVID-19 protects them from further infections and that they no longer need to be vaccinated. One respondent declared a lack of belief in the COVID-19 pandemic. Those who had contracted COVID-19 were significantly more likely to report an earlier SARS-CoV-2 infection as a reason behind their vaccination refusal (*p* = 0.050). Unmarried residents (32.1%) and those in marriages/partnerships without children (22.2%) more often indicated a willingness to wait for the effectiveness and long-term adverse effects of the vaccine to be assessed as their reason for potentially refusing vaccination (6.5%, *p* = 0.005). Moreover, those living alone more often indicated the following reasons for their reluctance to accept the vaccination, compared with those living with their family: a belief that the vaccine was not sufficiently tested (28.6%, 8.2%, and 19.4%, respectively; *p* = 0.045), a willingness to wait for the effectiveness and long-term adverse effects of the vaccine to be assessed (42.9%, 9.8%, and 22.2%, respectively; *p* = 0.005), and a fear of complications (14% vs. 5% of those living with the family, *p* = 0.022). There were no statistically significant correlations between the reasons for refusing the vaccine and gender, place of residence, having been in quarantine, or working with COVID-19 patients.

We analyzed the sources from which ophthalmology residents learned about the COVID-19 pandemic and the vaccination. Of the respondents, we discovered that 92.1% trust specialists in infectious diseases, virology, and epidemiology in terms of COVID-19 and vaccinations. Moreover, 63.5% of residents learned from the internet, and 13.5% from television and radio broadcasts; however, the obtained information from these sources was not from the abovementioned specialists. Furthermore, 50% of respondents declared that they read professional press regarding COVID-19 vaccination, and 4% declared that they read non-professional press relating to the aforementioned matter. For 19.8% of residents, friends and family were their sources of information about the pandemic. None of the respondents mentioned press conferences organized by the government or individual ministries as sources of information about the COVID-19 pandemic. There were no statistically significant correlations between the type of COVID-19 knowledge sources and gender, marital status, residence place, previous COVID-19 infection, having been in quarantine, or working with COVID-19 patients. A statistically significant relationship between COVID-19 acceptance and the use of individual sources of information on the pandemic has not been confirmed (Tables S1–S3 in Supplement). Multivariate logistic regression for willingness to take the COVID-19 vaccine is shown in Table 2.

## 4. Discussion

This study aimed to assess the attitudes of ophthalmology residents in Poland toward COVID-19 vaccination. Consequently, we created a questionnaire to collect the opinions of trainee ophthalmologists on COVID-19 vaccination. In our group of respondents, 71.4% of respondents declared that they will be vaccinated for COVID-19, 17.5% were unsure, and 11.1% were definitely against vaccination.

Surveys among various healthcare professionals in France indicated a slightly higher proportion of vaccine supporters (76.9%) [8]. The highest percentages of those wanting to become vaccinated were among physiotherapists (95.8%), doctors (92.1%), and pharmacists (88.8%), and the lowest were among nurses (64.7%) and assistant nurses (60.1%). If we compare our study group to the abovementioned group of French doctors, the differences to the disadvantage of Polish young medics are apparent. Other studies by French and French-speaking health professionals in Belgium and Canada found that 75% of French, 76% of Belgian, and 70% of Canadian HCWs want to get vaccinated for COVID-19 [6]. Vaccine acceptance studies in a group of healthcare professionals in the United States showed that 36% of HCWs were willing to get vaccinated as soon as possible, while 56% were not convinced to get vaccinated and wanted to wait to learn more about vaccination [5]. In contrast, in a study conducted in the Democratic Republic of Congo, only 27.7% of respondents expressed a willingness to get vaccinated for COVID-19 [9]. Attempting to objectively assess vaccination acceptance among HCWs in different countries is difficult, because the presented data cover heterogeneous groups of respondents and come from countries where the courses of the pandemic are different.

Our study confirms the findings of Szmyd et al. [10], who compared willingness to receive COVID-19 vaccination between two groups of health care workers: physicians and administrative healthcare assistants. In their study, 82.95% and 54.31% of participants from both groups were willing to get vaccinated, respectively. The main concern in both groups was the development of long-term adverse effects after receiving the COVID-19 vaccine [11]. Moreover, their study revealed that depression significantly affects one’s willingness to get vaccinated. Additional promptness was significantly strengthened by a positive medical history of recommended vaccinations, a fear of catching COVID-19, as well as a fear of passing on the disease to friends/relatives. The authors stated that, overall, the percentage of HCWs who want to be vaccinated against COVID-19 remained unsatisfactory.

We found that respondents living with families or close relatives were more eager to get vaccinated, as opposed to single respondents and respondents who were married or in relationships with children. In a study on French medics, older HCWs, especially men, were more willing to accept the vaccination than other respondents [8]. Among HCWs in the United States, vaccination acceptance increases with age, education, and income levels [5]. In contrast, those in rural areas, women, African Americans, and Hispanics were more likely to feel negatively about vaccination [5]. Males and physicians dominated a small proportion of the healthcare professionals who accepted vaccination in the Democratic Republic of Congo [9].

As this study shows, several factors simultaneously influence vaccination acceptance or rejection. The most common motivations for getting vaccinated for COVID-19 were a fear of infecting relatives and family, a belief in the effectiveness of the vaccine, a fear of self-infection, and a desire to set a good example for others. These reasons were most often given by people living with families and married people with children.

Residents who had contracted COVID-19 showed a similar desire to get vaccinated, compared to those who had not. However, one of the main reasons reported by such residents to receive the vaccine was that they would be able to benefit from the reduced epidemiological regime allowed for vaccinated individuals. The people that had contracted COVID-19 acquired, at least temporarily, natural immunity. In most cases, relatives were also simultaneously infected. Therefore, the fear of infection and transmitting the disease to the family is not the most important motivation for vaccination in such individuals. However, it should be emphasized that most of these people, despite having a certain immunity offered by their previous COVID-19 infection, want to get vaccinated as soon as possible. This may be due to their poor experience with SARS-CoV-2 and their uncertainty about the length of the immunization period. Our research has shown that people living alone and without children are less likely to want to be vaccinated. Such individuals may be less afraid of the pandemic, as most of these individuals are young, and are therefore less likely to develop symptoms.

Moreover, given the fact that they did not have to be afraid of transmitting the infection to close family members, their lower vaccination acceptance rate is understandable. The main reason for not vaccinating for COVID-19 among ophthalmology residents was a desire to have more knowledge on the effectiveness and long-term adverse effects of the vaccine. Moreover, there was a feeling of uncertainty regarding the vaccine’s safety. Evidence shows that the newer the vaccine, the greater the level of hesitation and non-acceptance of it [12]. A reason that vaccines typically pose little to no threat is the slow and methodical process of developing them, which can take up to several years. The accelerated approval of the new COVID-19 vaccines may raise doubts as to whether the vaccine has been adequately tested for safety and efficacy. It should also be noted that vaccination acceptance is greatly influenced not only by vaccine type, but also by the quality and availability of the healthcare system, as well as geographical, cultural, social, political, and emotional factors [13]. Another reason for hesitation may be the misinformation spread by anti-vaccine activists, who campaign against vaccines by spreading false information.

The analysis of sources from which residents of ophthalmology derive their knowledge regarding COVID-19 provides some interesting information. We have found that their confidence is not inspired by the data provided by government representatives or the Ministry of Health. In contrast, Verger et al. [6] found a link between a lack of trust in the health minister, in terms of his assurances about the safety of the COVID-19 vaccine, and the lower acceptance of vaccines. Negative attitudes toward vaccination among HCWs in the United States were also related to distrust in the government [5]. Our research shows that the vast majority of ophthalmology residents derive their knowledge regarding the COVID-19 pandemic and vaccination from infectious disease specialists, virologists, and immunologists, who speak unequivocally in favor of vaccination. Accordingly, the percentage of residents who are unsure about taking the vaccine, and that of residents who refuse vaccination (17.5% and 11.1%, respectively), is apparently high. It was found that, for many young healthcare professionals, theoretical considerations about vaccine safety—supported by clinical trials, but with a limited study group and a short follow-up period—do not provide a sufficient incentive for vaccination.

The sample size is the first thing that needs to be taken into account when analyzing the limitations of this study. This study covered a narrow group of medical professionals, with similar ages and experiences. This resulted in very homogeneous data, making the results are more reliable. Similar studies from other countries, also referring to other groups of doctors, could provide valuable comparative information. Moreover, the survey was conducted in early 2021, as the immunization program was just beginning. As the number of people vaccinated increases and new information about the efficacy and safety of the COVID-19 vaccine becomes available, the vaccination acceptance rate may increase. However, it is never possible to be “up to date” on this topic. Finally, some further studies on the COVID-19 infection rates among ophthalmologists should be considered; unfortunately, we do not have such data at this moment.

The vast majority of ophthalmology residents who took part in our survey demonstrated a willingness to be vaccinated against SARS-CoV-2. We assume that this number may not be representative of the general population, and this may be due to their greater health awareness. The willingness to get vaccinated as soon as possible may also be amplified by a fear of passing on the disease to relatives. Such fears may negatively influence the physician’s critical thinking and decision-making. On the other hand, the introduction of a program of vaccination against SARS-CoV-2 worldwide provides a certain perspective for the restoration of normality in many areas of life, perhaps most significantly in helping to relieve the psychological stress affecting both patients and HCWs [14]. Further studies providing insight into attitudes toward the COVID-19 vaccination among different populations and occupational groups are needed.

## 5. Conclusions

In summary, most ophthalmology residents expressed a willingness to get vaccinated for COVID-19. Marital status and cohabitation affect vaccination acceptance. Those who already had COVID-19 have different reasons for accepting or refusing vaccination. The number of residents who are unsure about taking the vaccine is significant; therefore, it is important to implement appropriate social promotion measures to attract this group of respondents for vaccination. Most of the respondents in our study learned about the pandemic from specialists in the field of viral diseases, with a complete lack of trust in politicians in this regard. Therefore, medical authorities should focus more on giving further knowledge regarding the COVID-19 pandemic, and demand and persuade citizens to take the COVID-19 vaccine. Reducing distrust regarding COVID-19 vaccination and increasing the willingness to take the vaccine among HCWs, may result in an increase in vaccination acceptance among the general population, which closely monitors how HCWs behave in this matter.

## Figures and Tables

**Table 1:** Sociodemographic characteristics of study participants.

Characteristic	Total Group	Female	Male	*p*
	*n* = 126 (%)	*n* = 102 (%)	*n* = 24 (%)	
Sex				
Female	102 (81.0)			
Male	24 (19.0)			
Marital status				
Single	53 (42.1)	44 (43.1)	9 (37.5)	0.590
Married with kids	46 (36.5)	38 (37.3)	8 (33.3)	
Married without kids	27 (21.4)	20 (19.6)	7 (29.2)	
Living with				
Family	61 (48.4)	49 (48.0)	12 (50.0)	0.808
Partner	36 (28.6)	1 (1.0)	-	
Friends	1 (0.8)	28 (27.5)	8 (33.3)	
Single	28 (22.2)	24 (23.5)	4 (16.7)	
Place of living				
Village	9 (7.1)	8 (7.8)	1 (4.2)	0.329
City of up to 50k citizens	6 (4.8)	3 (2.9)	3 (12.5)	
City of 50–150k citizens	19 (15.1)	16 (15.7)	3 (12.5)	
City of 150–500k citizens	35 (27.8)	27 (26.5)	8 (33.3)	
City of over 500k citizens	57 (45.2)	48 (47.1)	9 (37.5)	
Hospital ward				
Treating COVID-19 patients	42 (33.3)	35 (34.3)	7 (29.2)	0.810
Not treating COVID-19 patients	84 (66.7)	67 (65.7)	17 (70.8)	

Males and females compared using the chi-square test or Fisher’s exact test. k: thousands, COVID-19: coronavirus disease of 2019.

**Table 2:** Multivariate logistic regression for willingness to take the COVID-19 vaccine.

	OR	95% CI for OR	*p*
Sex, Male	1.80	0.61–6.16	0.312
Marital status (single = reference)			
Married with kids	1.41	0.27–7.11	0.678
Married without kids	0.31	0.09–1.04	0.064
Living with (family = reference)			
Partner / Friends	1.003	0.24–4.03	0.997
Single	0.37	0.07–1.78	0.220
Place of living (village/city of up to 50k citizens = reference)			
City of 50–150k citizens	0.42	0.05–2.51	0.368
City of 150–500k citizens	0.51	0.07–2.60	0.454
City of over 500k citizens	0.49	0.06–39	0.414
Hospital ward, not treating COVID-19 patients	0.47	0.17–1.21	0.132

OR: odds ratio, with 95% confidence interval (CI). None of the sociodemographic variables proved to significantly predict willingness to take the COVID-19 vaccine, as per the logistic regression model.

## Data Availability

Readers can access the data supporting the conclusions of the study upon email request. The names and personal data of the participants cannot be released, due to ethical aspects.

## Supplementary Materials

The following are available online at https://www.mdpi.com/article/10.3390/vaccines9040371/s1, Supplement: The Respondent s Survey, Table S1. Attitudes toward COVID-19 vaccines be-tween males and females. Table S2. Attitudes toward COVID-19 vaccines between respond-ents working and those not working with COVID-19 patients. Table S3. Attitudes toward COVID-19 vaccines vs. having been tested against COVID-19.
