# Supplementary material for: Intention to Get COVID-19 Vaccinations among Ophthalmology Residents in Poland: A Cross-Sectional Survey
Source: Vaccines (Basel). 2021 Apr 11;9(4):371. doi: 10.3390/vaccines9040371 (PMC8070351; doi:10.3390/vaccines9040371)
Supplement: Supplementary file 1 [file vaccines-09-00371-s001.pdf]

Table S1. Attitudes toward COVID-19 vaccines between males and females.

Characteristic	Total group	Female	Male	*p*
N	126	102	24	
Q11: Would you take a COVID vaccine, if possible?				
Yes	90 (71.4)	71 (69.6)	19 (79.2)	
No	14 (11.1)	12 (11.8)	2 (8.3)	0.713
Don't know	22 (17.5)	19 (18.6)	3 (12.5)	
Q12: If you would not take a COVID vaccine, why?				
Fear of complications	15 (11.9)	13 (12.7)	2 (8.3)	0.734
Vaccine is not checked	21 (16.7)	17 (16.7)	4 (16.7)	>0.999
I do not believe in effectiveness of vaccine	2 (1.6)	1 (1.0)	1 (4.2)	0.346
I had COVID so I do not need to take a vaccine	15 (11.9)	13 (12.7)	2 (8.3)	0.734
I do not believe in COVID	1 (0.8)	1 (1.0)	-	>0.999
I will wait for assessment of effectiveness and far side effects of vaccine	26 (20.6)	19 (18.6)	7 (29.2)	0.386
I am against any vaccines	-	-	-	-
Other	-	-	-	-
Q13: If you would take a COVID vaccine, why?				
I believe in vaccine effectiveness	70 (55.6)	57 (55.9)	13 (54.2)	>0.999
I am afraid of being infected	63 (50.0)	50 (49.0)	13 (54.2)	0.821
I am afraid of infecting my family	83 (65.9)	64 (62.7)	19 (79.2)	0.198
I want to give a good example	62 (49.2)	48 (47.1)	14 (58.3)	0.443
I am not afraid of complications	23 (18.3)	18 (17.6)	5 (20.8)	0.944
I count on some ease in epidemiologic regime for vaccinated people	39 (31.0)	32 (31.4)	7 (29.2)	>0.999
Other	4 (3.2)	3 (2.9)	1 (4.2)	0.575
Q14: Sources of knowledge on COVID				
Statements by epidemiologists, virologists and infectious diseases' specialists	116 (92.1)	95 (93.1)	21 (87.5)	0.401
The Internet	80 (63.5)	63 (61.8)	17 (70.8)	0.552
Government press conferences	-	-	-	-
TV, radio	17 (13.5)	13 (12.7)	4 (16.7)	0.740
Specialist press, incl. foreign	63 (50.0)	52 (51.0)	11 (45.8)	0.821
Non-specialist press	5 (4.0)	5 (4.9)	-	0.582
Friends	25 (19.8)	23 (22.5)	2 (8.3)	0.158

Data are presented as *n* (% of group). Males and females were compared using chi-square tests or Fisher's exact tests.

Table S2. Attitudes toward COVID-19 vaccines between respondents working and those not working with COVID-19 patients.

Characteristic	Total group	COVID group	Non-COVID group	*p*
N	126	42	84	
Q11: Would you take a COVID vaccine, if possible?				
Yes	90 (71.4)	33 (78.6)	57 (67.9)	
No	14 (11.1)	2 (4.8)	12 (14.3)	0.278
Don't know	22 (17.5)	7 (16.7)	15 (17.9)	
Q12: If you would not take a COVID vaccine, why?				
Fear of complications	15 (11.9)	4 (9.5)	11 (13.1)	0.772
Vaccine is not checked	21 (16.7)	6 (14.3)	15 (17.9)	0.799
I do not believe in effectiveness of vaccine	2 (1.6)	1 (2.4)	1 (1.2)	>0.999
I had COVID so I do not need to take a vaccine	15 (11.9)	4 (9.5)	11 (13.1)	0.772
I do not believe in COVID	1 (0.8)	-	1 (1.2)	>0.999
I will wait for assessment of effectiveness and far side effects of vaccine	26 (20.6)	7 (16.7)	19 (22.6)	0.586

Characteristic	Total group	COVID group	Non-COVID group	*p*
I am against any vaccines	-	-	-	-
Other	-	-	-	-
Q13: If you would take a COVID vaccine, why?				
I believe in vaccine effectiveness	70 (55.6)	24 (57.1)	46 (54.8)	0.949
I am afraid of being infected	63 (50.0)	22 (52.4)	41 (48.8)	0.850
I am afraid of infecting my family	83 (65.9)	28 (66.7)	55 (65.5)	>0.999
I want to give a good example	62 (49.2)	19 (45.2)	43 (51.2)	0.659
I am not afraid of complications	23 (18.3)	5 (11.9)	18 (21.4)	0.289
I count on some ease in epidemiologic regime for vaccinated people	39 (31.0)	13 (31.0)	26 (31.0)	>0.999
Other	4 (3.2)	-	4 (4.8)	0.300
Q14: Sources of knowledge on COVID				
Statements by epidemiologists, virologists and infectious diseases' specialists	116 (92.1)	38 (90.5)	78 (92.9)	0.907
The Internet	80 (63.5)	25 (59.5)	55 (65.5)	0.647
Government press conferences	-	-	-	-
TV, radio	17 (13.5)	5 (11.9)	12 (14.3)	0.927
Specialist press, incl. foreign	63 (50.0)	22 (52.4)	41 (48.8)	0.850
Non-specialist press	5 (4.0)	3 (7.1)	2 (2.4)	0.332
Friends	25 (19.8)	8 (19.0)	17 (20.2)	>0.999

Data are presented as n (% of group). Respondents working and those not working with COVID-19 patients were compared using chi-square tests or Fisher's exact tests.

Table S3. Attitudes toward COVID-19 vaccines vs. having been tested against COVID-19.

Characteristic	No COVID test	COVID test, negative	COVID test, positive	*p*
N	28	72	26	
Q11: Would you take a COVID vaccine, if possible?				
Yes	17 (60.7)	52 (72.2)	21 (80.8)	
No	3 (10.7)	8 (11.1)	3 (11.5)	0.383
Don't know	8 (28.6)	12 (16.7)	2 (7.7)	
Q12: If you would not take a COVID vaccine, why?				
Fear of complications	5 (17.9)	7 (9.7)	3 (11.5)	0.521
Vaccine is not checked	7 (25.0)	11 (15.3)	3 (11.5)	0.393
I do not believe in effectiveness of vaccine	-	1 (1.4)	1 (3.8)	0.419
I had COVID so I do not need to take a vaccine	5 (17.9)	7 (9.7)	3 (11.5)	0.521
I do not believe in COVID	-	1 (1.4)	-	>0.999
I will wait for assessment of effectiveness and far side effects of vaccine	7 (25.0)	16 (22.2)	3 (11.5)	0.432
I am against any vaccines	-	-	-	-
Other	-	-	-	-
Q13: If you would take a COVID vaccine, why?				
I believe in vaccine effectiveness	12 (42.9)	44 (61.1)	14 (53.8)	0.252
I am afraid of being infected	17 (60.7)	34 (47.2)	12 (46.2)	0.436
I am afraid of infecting my family	20 (71.4)	49 (68.1)	14 (53.8)	0.331
I want to give a good example	15 (53.6)	33 (45.8)	14 (53.8)	0.682
I am not afraid of complications	4 (14.3)	14 (19.4)	5 (19.2)	0.903
I count on some ease in epidemiologic regime for vaccinated people	9 (32.1)	16 (22.2)	14 (53.8)	0.011
Other	-	4 (5.6)	-	0.492
Q14: Sources of knowledge on COVID				
Statements by epidemiologists, virologists and infectious diseases' specialists	23 (82.1)	68 (94.4)	25 (96.2)	0.117
The Internet	21 (75.0)	42 (58.3)	17 (65.4)	0.322
Government press conferences	-	-	-	-

Characteristic	No COVID test	COVID test, negative	COVID test, positive	*p*
TV, radio	5 (17.9)	6 (8.3)	6 (23.1)	0.115
Specialist press, incl. foreign	12 (42.9)	39 (54.2)	12 (46.2)	0.542
Non-specialist press	2 (7.1)	2 (2.8)	1 (3.8)	0.595
Friends	6 (21.4)	14 (19.4)	5 (19.2)	0.954

Data are presented as *n* (% of group). Groups were compared using chi-square tests or Fisher's exact tests.
